# Enhancing Nutrient Profile and Reducing Acrylamide in California-Style Table Olives with *Cassia grandis* Fortification

**DOI:** 10.3390/foods14081426

**Published:** 2025-04-21

**Authors:** Ismael Montero-Fernández, Víctor Manrique Fernández, Francisco Pérez-Nevado, Selvin Antonio Saravia-Maldonado, Jhunior Abraham Marcía Fuentes, Daniel Martín-Vertedor

**Affiliations:** 1 Department of Plant Biology, Ecology and Earth Sciences, Faculty of Science, Universidad de Extremadura, Avda. de Elvas, s/n, 06071 Badajoz, Spain; 2 Research Institute of Agricultural Resources (INURA), Campus Universitario, Avda. de la Investigación, s/n, 06006 Badajoz, Spain; fpen@unex.es (F.P.-N.); daniel.martin@juntaex.es (D.M.-V.); 3 School of Agricultural Engineering, Universidad de Extremadura, 06007 Badajoz, Spain; vimanriqu@alumnos.unex.es; 4 Faculty of Earth Sciences and Conservation, Universidad Nacional de Agricultura, Catacamas 16201, Honduras; saraviaselvin@yahoo.com; 5 Doctoral Program in Sustainable Territorial Development, Science Faculty, Universidad de Extremadura, 06007 Badajoz, Spain; 6 Faculty of Technological Sciences, Universidad Nacional de Agricultura Road to Dulce Nombre de Culmi, Km 215, Barrio El Espino, Catacamas 16201, Honduras; jmarcia@unag.edu.hn; 7 Aquaculture Center ‘Las Vegas del Guadiana’, Regional Government of Extremadura, N-5, km 391.7, Villafranco del Guadiana, 06195 Badajoz, Spain

**Keywords:** minerals, antioxidants, liquid pressurization, nutrients, enriched foods

## Abstract

In this study, Californian-style black table olives were enriched with fresh and lyophilized “Carao” (*Cassia grandis* L.) to enhance their mineral composition, antioxidant activity, phenolic compound content, and to evaluate their potential for reducing acrylamide levels. Mineral concentrations were quantified using inductively coupled plasma optical emission spectrometry (ICP-OES). The addition of both fresh and lyophilized “Carao” significantly increased the iron concentration in the olives. Additionally, levels of calcium, magnesium, and potassium were elevated in both “Carao” treatments. Among the treatments, the addition of fresh “Carao” resulted in the highest increase in antioxidant activity, followed by the lyophilized “Carao”, with increases of 62.3% and 68.3%, respectively. The effect of fresh and lyophilized “Carao” on acrylamide reduction in oxidized olives is also discussed.

## 1. Introduction

The olive tree (*Olea europea* L.) is closely linked to Mediterranean culture where it has been cultivated and consolidated for more than 6000 years and its fruit has been consumed either as oil or as table olives [1]. The production of table olives in Spain in the last year was 62,000 t [2]. Within the Mediterranean countries of southern Europe, particularly in Spain and Italy, Spain is one of the main countries in the olive industry, accounting for more than half of the total cultivation area, followed by Portugal, Greece, France, Croatia, and Italy [3]. Spanish, Californian, and natural-style olives are the most common types of table olives worldwide. However, the Californian-style is one of the most used procedures [4,5]. In the elaboration process at the industrial level, olives are immersed in lye solutions, with or without air bubbling to cause darkening via oxidation and then packaged with governing liquid that contains salt solution, calcium chloride, and ferrous gluconate. Afterwards, the sterilization process (121–126 °C, 15–30 min) is carried out to microbiologically stabilize the product [6]. Heat treatment of food leads to the formation of toxic organic compounds that are highly harmful to health, such as heterocyclic aromatic amines and acrylamide [7]. Acrylamide is a known carcinogenic compound that is formed during the heating of foods rich in reducing sugars and asparagine [8], which is currently recognized by the European Union in different regulations [9,10,11]. 

Food fortification is a process in which specific nutrients are added to foods to address deficiencies. These nutrients can be added either through direct addition or by encapsulation [12,13]. Through fortification, the nutritional density of foods that are easily consumed is therefore improved and can be applied to different food matrices, such as fresh vegetables, seaweed, concentrated nutritional powders, pulverized vegetables, or liquid extracts such as proteins or milk [14]. Also, modern biotechnological practices are employed in fortification processes to increase the levels of micronutrients, such as vitamins and minerals [15].

Foods rich in bioactive molecules have increasingly gained importance as healthy additives and natural supplements to support immune health [16]. Furthermore, there is an increasing demand for functional foods valued in 2019 at USD 177,770.0 and it is estimated to reach USD 267,924.4 million in 2027, registering an increase in the consumption of these foods of 6.7% [17]. Among the bioactive compounds, phenolic compounds, vitamins, fatty acids, terpenes, and minerals stand out.

*Cassia grandis* is a fruit from Central America, the Caribbean, and Northern South America, reaching 30 m in height [18]. Both the plant and the fruit of *C. grandis* have traditionally been used for different purposes due to their biological potential, such as the treatment of anemia and skin ulcers [19]. In addition, it is a plant that has antioxidant [20,21] and analgesic activity [20]. Regarding its chemical composition, there are flavonoids, phenols, coumarins, saponins, and triterpenes, among other compounds [20]. It is also rich in volatile organic compounds such as 3-methyl-butanoic acid, 2-methyl-butanoic acid, benzaldehyde, and L-α-terpineol [20], being therefore a fruit that can be used in food fortification. One of the uses of “Carao” is in the fortification of yogurt, since it increases the health benefits without producing major changes in the sensory, organoleptic characteristics, and properties of yogurt, as well as does not influence microbial growth such as *Streptococcus thermophilus* and *Lactobacillus bulgaricus* [22]. The production of camel milk yogurt with the addition of “Carao” has also been experimented with to evaluate the increase in probiotic characteristics since it improves the anti-inflammatory stimulus and antioxidant activity in HT-29 cells. In addition, “Carao” pulp is a rich source of minerals such as iron and zinc, which is why it has been traditionally used to combat anemia [23].

Polyphenols are a relevant group of secondary metabolites since they present biological activities that are beneficial to health such as their antitumor potential, as a neuroprotective agent in the brain, anti-inflammatory effect, or due to their antimicrobial actions [24]. It has also been shown that these compounds can reduce acrylamide levels in foods due to their antioxidant effect [25,26]. Certain polyphenols found in fruits, vegetables, red wine, and tea can prevent the production of acrylamide during cooking since they can capture free radicals and chelate metal ions that contribute to the formation of acrylamide since the toxicity of acrylamide is mainly caused by oxidative stress and the generation of free radicals [27,28]. As previously indicated, “Carao” is a fruit known for its high content of bioactive compounds, such as polyphenols and others. Given that table olives are a staple in the Mediterranean diet, the development of “Carao” fortified table olives presents a valuable opportunity to enhance their nutritional profile. In the case of Californian-style table olives, they may offer protective benefits by reducing the formation of acrylamide during the processing stage.

The objective of this work was to fortify Californian-style table olives to enhance their mineral composition, antioxidant activity, and phenolic compound content. Additionally, the reduction in acrylamide levels in olives after the addition of “Carao” was also evaluated.

## 2. Materials and Methods

### 2.1. Samples

A total of 100 kg of fresh “Carao” at optimal ripening was collected in the “Guanipol Reserve” (Honduras). The pulps were manually separated from the rest of the fruit. One part of the pulp was lyophilized in a lyophilizer (Virtis Company, Gardiner, NY, USA, Mod. Génesis 25 LL, Hücoa-Herlos) and vacuum-packed, while the other part was dried in an oven with air circulation (Digitronic-TFT) for 48 h at 50 °C. Subsequently, it was ground in a paddle mill until obtaining a particle size of 501–700 µm. Samples were vacuum-packed (Gustav Müller VS 100, Maintal, Germany) in bags and stored under frozen conditions (−80 °C) until extraction processes were carried out.

Olives (*Olea europaea* L.) of the “Hojiblanca” variety (100 kg) were harvested in a green state of ripeness in a test field at the CICYTEX research center (Badajoz, Spain) during the 2022/23 season, being stored in brine until the analysis was carried out.

### 2.2. Californian-Style Table Olives Elaboration Process

**Figure 1:**
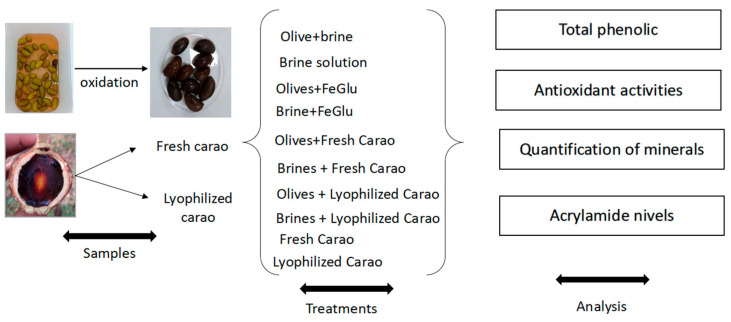
Diagram of the experimental design.

The diagram of the experimental design is shown in Figure 1. The stored olives were transformed into California-style black olives following the method described by Martín-Vertedor et al. [29], where they were treated with NaOH until it penetrated the seeds of the olives, being subsequently washed and neutralized with lactic acid solution (80% *v*/*v*) and bubbling with carbon dioxide so that their oxidation occurred. To fix their black color, a solution of ferrous gluconate (FeGluc) (0.15%) was added for 4 h and then removed (Panreac, Barcelona, Spain). “Carao” pulp was added to Californian-style table olives; eight different treatments were performed: (i) Olive + brine, (ii) Brine solution, (iii) Olives + FeGlu, (iv) Brine + FeGluc, (v) Olives + Fresh “Carao”, (vi) Brines + Fresh “Carao”, (vii) Olives + Lyophilized “Carao”, and (viii) Brine + Lyophilized “Carao”. Also, two control treatments were carried out: (ix) Fresh “Carao” and (x) Lyophilized “Carao”. The amounts of “Carao” added were all 5% *w*/*w*. Three replicates per sample were prepared. After that, they were placed in cans (150 g) with a new brine solution containing 2% *w*/*v* of sodium chloride solution (Panreac, Barcelona, Spain), ferrous gluconate (0.15% *w*/*v*), and 2 g L^−^^1^ CaCl_2_ (*w*/*v*) (Tetra Chemicals Europe, Helsingborg, Sweden). Samples were submitted to a sterilization process using an industrial autoclave with an accumulated lethality of 15 min inside the can [4,6]. After the sterilization process, cans were stored at room temperature until analysis.

Samples were taken in triplicate and analyses were performed for total phenols, antioxidant activity, mineral quantification, and acrylamide levels.

### 2.3. Total Phenolic Compounds and Antioxidant Activities

For the determination of total phenolic compounds in the controls and in the eight different treatments of olives and brines, the Folin–Ciocalteu method was used according to the methodology described by Swain et al., 1959 [30] and Brand-Williams et al., 1995 [31]. For this purpose, methanolic extracts were prepared for each of the treatments in a ratio (2:1), which were subjected to mechanical agitation for 24 h and subsequently centrifuged for 10 min at 3500 rpm. From the supernatant, 250 μL and a standard gallic acid solution were taken as a positive control. Then, 15 mL of distilled water and 1.25 mL of the Folin–Ciocalteu reagent were added to each of the samples. The homogenized samples were allowed to rest for 8 min in the dark and 3.75 mL of 7.5% sodium carbonate solution was added and brought to a volume of 25 mL with distilled water. The flasks were homogenized and kept in the dark for two hours, with absorbance readings being measured at 765 nm. Phenolic compounds analysis was performed in triplicate.

For the measurement of antioxidant activity, the DPPH reduction method was used according to the methodology described by Martín-Vertedor et al., 2021 [4], wherein a standard DPPH solution of 0.2 mg mL⁻^1^ in methanol was prepared. From the extracts prepared for the determination of total phenolic compounds, 0.3 mL was taken for each of the treatments and 1 mL of the standard DPPH solution was added to each of the treatments, recording the absorbance at 517 nm. The other method used for antioxidant activity was the ABTS method, according to the method described by Liu et al. [32].

For the ABTS method, the ABTS^•+^ radical was generated by reaction of a stock solution of 7 mM 2,2′-azinobis-(3-ethylbenzothiazolin-6-sulfonic acid) in 1 mL of 2.45 mM potassium persulfate stored for 12 h before use. Subsequently, 150 µL of the extracts was taken and diluted in 14 mL of buffer phosphates (8 g NaCl, 0.2 g of KCl and 1.44 g of KH_2_PO_4_) contained in 1 L of distilled water until obtaining an absorbance of 0.700 at a wavelength of 734 nm. The extracts were prepared by reacting 100 mL of each of the samples with 1000 mL of ABTS^•+^, measuring the absorbance at 734 nm. Antioxidant activity analysis was performed in triplicate.

### 2.4. Acrylamide Analysis

The determination of the acrylamide content was carried out following the extraction methodology proposed by Fernández et al. [26], where 10 mL of water was added to 2 g of one of two treatments; one of them was brine and the other was olives with “Carao”, since acrylamide is significantly high in the governing liquid of olives. These analyses were carried out, homogenizing for 1 h and centrifuging at 1677 rpm at 4 °C. The extract was filtered with 0.22 µm filters, determining the acrylamide content by LC-MS (Agilent Technologies, Palo Alto, CA, USA). The injection volume was 3 μL and the separation was carried out using a reversed-phase system with a Zorbax XDB-C18 HPLC column (3.5 µm, 150 mm × 2.1 mm). It was operated isocratical at a temperature of 30 °C composed of 95% of solvent A (0.1% formic acid in Mili-Q water) and 5% of solvent B (0.1% formic acid in methanol). The flow rate was 0.25 mL min^−1^. Detection was performed with an Agilent Technologies 6460 triple–quadrupole mass spectrometer equipped with an electrospray ion source operating in positive ion mode.

### 2.5. Micronutrients Analysis with ICP-OES

The minerals were analyzed following the methodology proposed by Lodolini et al. [33]. To perform the mineral analysis, 2 g of sample of each of the treatments was dried at 100 °C for 24 h and incinerated in a muffle furnace at 550 °C (1 °C min^−1^). The ashes were dissolved in 20 mL of 4% HNO_3_. The mineral content was determined using an ICP-OES (Perkin-Elmer 5300 DV model) in radial mode, by injecting 3 µL and operating with a concentric nebulizer and a cyclonic nebulization chamber, with the results expressed in mg kg^−1^.

### 2.6. Statistical Analysis

A one-way ANOVA was performed to establish significant differences between the different established experimental treatments. Tukey’s multiple range test was performed to know which treatments differ from others at *p* < 0.05. SPSS 18.0 software was used for statistical analysis (SPSS Inc., Chicago, IL, USA). Data are expressed as means and standard deviations (SDs).

## 3. Results and Discussion

### 3.1. Total Phenolic Compounds and Antioxidant Activities

The values of total phenolic compounds and antioxidant activities are shown in Table 1. Total phenolic compounds (TPCs), expressed as mg 100 g^−1^ of GAE, presented higher values for fresh “Carao” pulp followed by lyophilized “Carao”. Fresh “Carao” has 12.7% TPCs compared to lyophilized “Carao”. This fact may be due to the degradation that these compounds may have suffered in the freeze-drying process. Of the treatments studied, those with the highest TPCs content were Olives + Fresh “Carao” followed by Olive + Lyophilized “Carao”. These treatments presented 78.2% more TPCs compared to Olives Brines for Olives + Fresh “Carao” and 27.1% for Olive + Lyophilized “Carao”. It is also observed that phenolic compounds pass from “Carao” to brines since, when these are in the presence of “Carao”, they considerably increase the concentration of TPCs. With these results, the viability of fortifying table olives by developing foods with added value is evident, since polyphenols are considered one of the main food ingredients in the human diet due to their antioxidant properties and other types of bioactivities such as anti-inflammatory, antiallergic, antidiabetic, anticancer, and vasodilators [34,35].

The antioxidant activities measured by the 2,2′-azino-bis-3-ethylbenzthiazoline-6-sulphonic acid (ABTS) and 1,1-diphenyl-2-picrylhydrazyl (DPPH) method were slightly different for each of the two methods [36] point out that the antioxidant capacity obtained by two different methods is different, being related to the chemical structure of the phenolic compounds involved with said activities. It is observed that antioxidant activities are slightly higher using the ABTS method in relation to DPPH. The highest values of antioxidant activity were found in fresh “Carao” followed by lyophilized ‘Carao’. Of all the treatments studied, the one that presented the highest values of antioxidant activities was Olives + Fresh “Carao” since in said treatment phenolic compounds are present on the part of the table olive and on the part of “Carao”. In this treatment, antioxidant activities increased by 62.3% in relation to the Olive + Brine treatment using the DPPH method and by 68.4% when the ABTS method was used. The next treatment that produced high values of antioxidant activities was “Olives lyophilized”, whose increase in antioxidant capacity compared to the Olives + Brine treatment which was 33.0% using the DPPH method and 52.7% using the ABTS method. The values are always higher using the ABTS method, since authors such as Floegel et al. [37] point out that, using this technique, higher values are obtained when applied to foods that contain hydrophilic, lipophilic, and pigmented compounds. Finally, it should be noted that in the “brine solution” treatment there is a decrease in antioxidant activities in relation to any of the treatments. With these results, it can be stated that both fresh and lyophilized “Carao” is a good source of bioactive products with high antioxidant activity that can be used as a supplement in other foods such as table olives.

### 3.2. Determination of Minerals in California-Style Table Olives After the Addition of “Carao” Pulp

Table 2 shows the composition of different micronutrients for both fresh and lyophilized “Carao”, as well as for the eight treatments carried out (Olives + brine; brine solution; Olives + FeGluc; Brine + FeGluc; Olives + Fresh “Carao”; Brine + Fresh “Carao”; Olives + Lyophilized “Carao” and Brine + Lyophilized “Carao”).

As can be seen, the micronutrient that appears in the highest concentration in “Carao” pulp is Fe, producing a loss of 36.2% of this mineral when undergoing the freeze-drying process in relation to “Fresh Carao” (3294.7 ± 121.6 mg kg^−1^). Iron is found in low concentrations in the brine at 57.0 ± 6.4 mg kg^−1^ compared to the other treatments, corresponding to a contribution of this mineral from the olive at 63.4 mg kg^−1^. By adding ferrous gluconate to table olives, there is a considerable increase in the concentration of iron, especially in the brine, since it is a highly soluble compound in the brine and provides a high concentration of iron, in its soluble form as Fe^2+^ which is what can be absorbed by the body [38].

This effect is also reflected in the treatments to which “FeGluc” is added compared to the addition of “fresh or lyophilized Carao”, where the quantified iron concentration is higher. Although olives are being enriched with iron, for that iron to be available for absorption by the body, it must be in the reduced form (Fe^2+^), since when it is oxidized to Fe^3+^, it has difficulty being absorbed by the gastrointestinal tract [39,40]. One of the techniques used to help iron absorption is to form a chelate with ascorbic acid, since this compound has the ability to reduce and chelate iron compounds, so that their solubility and absorption increases at alkaline pH in the duodenum [37]. It should be noted that, in the treatments in which the amount of iron has been measured in olives in which “Carao” has been added, both in its fresh and lyophilized form, there have been no significant differences in iron concentrations.

Zinc was another micronutrient found in its highest concentration after the addition of iron, whose concentrations present in fresh and lyophilized “Carao” were of 13.6 ± 8.6 mg kg^−1^ for fresh “Carao” and 12.5 ± 3.5 mg kg^−1^ for lyophilized “Carao”. The “Olives + brine” treatment has a zinc concentration of 10.9 ± 2.4 mg kg^−1^, slightly lower than that found in “fresh” and “lyophilized Carao”. This micronutrient is very soluble in the brine solution, slightly increasing the concentration of zinc and the one that provides the greatest amounts of this micronutrient is FeGluc, reaching concentrations of 19.0 ± 3.3 mg kg^−1^ for this treatment. When the fresh and lyophilized “Carao” was added to the olives, it was again more soluble in the brines than in the olive, increasing by 27.7% in the “Olives + Fresh Carao” treatment in relation to the “Brine + Fresh Carao” treatment and increasing also by 13.2% for the “Olives + Lyophilized Carao” treatment compared to the “Brine + Lyophilized Carao” treatment. On the other hand, the increase in zinc concentration in the “Olive + Fresh Carao” treatment in relation to the “Olive + Brine” treatment is of 2.7% and in the “Olive + Lyophilized Carao” treatment compared to “Olives + Brines” it is of 7.6%, a lower absorption than for iron. This trace element is essential as a catalyst for cellular metabolism in addition to acting as a structural element and regulator of gene expression, also intervening in normal physical growth, in the process of neuron transduction and in reproductive function [41,42]. Since the body can mobilize only a small amount of endogenous zinc for metabolism, an adequate daily intake of this micronutrient is important to maintain physiological functions. The bioavailable sources of zinc in the body mostly come from animal origin, while the zinc content in plant foods depends on the concentrations in which it is found in the soil, so post-harvest food fortification is important to guarantee the intake of necessary micronutrients [43], but the concentrations of this element in foods must be controlled since abnormal concentrations of zinc ions in the body can develop serious consequences for the body such as neurodegenerative diseases or immune diseases [44]. The required doses of this micronutrient are 14–10 mg day^−1^ for adults [45]. Manganese, another important micronutrient in the diet and involved in functions such as the production and expression of oxidoreductases and manganese superoxide dismutase (MnSOD) [43], was found in concentrations higher than those of zinc in “fresh Carao” (18.2 ± 5.4 mg kg^−1^) and lyophilized (13.6 ± 3.6 mg kg^−1^), but both the olives and the brines present concentrations of this metal lower than 0.5 mg kg^−1^ and only the absorption of this micronutrient occurred for the brines both with the addition of “fresh Carao” (5.0 ± 2.1 mg kg^−1^) and with the addition of “lyophilized Carao” (7.4 ± 5.4 mg kg^−1^). The last of the micronutrients, copper, was quantified in concentrations less than 0.5 mg kg^−1^ in both “fresh” and “lyophilized Carao” and in all treatments. This micronutrient, like zinc, is essential for the development of biochemical and physiological functions, although it has antagonistic effects with zinc since the absorption of this element can suppress both the absorption of copper and iron [46,47].

Other micronutrients quantified were calcium (Ca), magnesium (Mg), and potassium (K). A slight loss of these nutrients is observed with the “freeze-drying” process, except for Ca, whose loss during “freeze-drying” was almost 75%. Of the four studied, the two most important are Ca and K. Ca is present in the olive in a concentration of 556.8 ± 25.6 mg kg^−1^, in a higher concentration than in the brine. By adding “Carao” to olives, the concentration of Ca increases approximately 2.5 times more than without adding “Carao”. The concentration of Ca also increases in the brine when both fresh and lyophilized “Carao” are added. By adding “fresh Carao” to the brine, the Ca concentration increases 8 times more than without adding “Carao”, and for “lyophilized Carao” the Ca concentration in the brine increases 6 times more than without adding “Carao”. With this implementation of the Ca levels in the olive after of the addition of “Carao”, the problem of Ca deficiencies in the diet worldwide can be corrected, since estimates indicate that approximately half of the world’s population has access to Ca. Ca is consumed in inadequate amounts in the diet, especially in developing countries [48,49]. This element is crucial in the formation of bones in addition to maintaining the balance of intracellular and extracellular fluids [50]. The daily Ca recommendations according to the WHO [51] in adult men is 1000 mg day^−1^ and, from 70 years of age, 1200 mg day^−1^, the same as for women. Adolescents who are of growing age require a calcium concentration of 1300 mg day^−1^ and for children between 4 and 8 years old, the recommended amounts of Ca are 1000 mg day^−1^, so the addition of “Carao” to olives of the Californian-style kind could help improve daily intake levels of this element.

Potassium is the major element found in “Carao” with concentrations of 2590.6 ± 28.3 mg kg^−1^ for “fresh Carao” and 2103.4 ± 22.3 mg kg^−1^ for “lyophilized Carao”. This concentration is high in “Olives + brine” at 1094.3 ± 154.2 mg kg^−1^ and increases by 30.3% when FeGlu is added. Also, the K concentration increases by 54.3% in the “Olives + Fresh Carao” treatment and by 33.86% for the “Olives + Lyophilized Carao” treatment. The amounts of this nutrient are important in the body, being the most exchangeable and abundant cation in the body. It is involved in various metabolic functions such as by acting as an electrolyte, being involved in the maintenance of blood pressure, glucose uptake, muscle contraction, impulse transmission, and various protein syntheses [52,53,54]. Daily recommended doses of this element are 3400 mg for adult men and 2600 mg for women. In adolescents, the required doses of K are 2300 mg for girls and 3000 mg for boys, and 400 mg daily for babies, according to the National Institute of Health [55]. These recommendations are based on the levels recommended by the World Health Organization, which are based on direct evidence that increases in potassium intake are associated with reductions in systolic and diastolic blood pressure [51]. Potassium levels in the body are rarely found in low concentrations, except in hospitalized patients who, due to diseases such as chronic diarrhea that leads to hypokalemia, have potassium levels which are never below 390 mg day^−1^ [56].

Magnesium is another of the important nutrients for the body, highlighting its crucial role in the metabolism of insulin regulation and glucose metabolism [57]. Since the ratio of Ca and Mg in foods is 2:1, the recommended intake of Ca in the body is between 1.70 and 2.60 [58]. When low magnesium intakes are combined with high calcium intakes, there is an increased risk of suffering from cardiovascular diseases, tumor diseases, and abnormal levels of vitamin D, among others [59]. In table proportion, the olive has a magnesium concentration of 247.3 ± 14.15 mg kg^−1^, and this element in the olive is related to Ca (2:1). Both brine and FeGluc provide relatively low Mg concentrations < 0.5 mg kg^−1^ to the olive. By adding FeGluc to the olives, the Ca concentration only increases compared to “olive + brine” by 21.31%. After the addition of fresh and lyophilized “Carao” to the olive, the concentration of Mg is absorbed in greater quantities in the brine than in the olive, increasing the concentration of Mg in the olive after adding fresh “Carao” by 125% and after adding lyophilized “Carao” by 109%, so the contribution of Mg to the olive is considerable. Finally, it should be noted that the contribution of Na to the olive by “Carao” is not significant, since this element in both the “fresh Carao” and the “lyophilized Carao” is found in lower concentrations in relation to the olive, Na being the element found in majority in it, with concentrations of 1463.2 ± 254.5 mg kg^−1^. Sodium is an element that, if consumed in excess, increases the risk of suffering from hypertension and cardiovascular diseases [60].

### 3.3. Influence of “Carao” Addition in the Synthesis of Acrylamide in Californian-Style Table Olives

**Figure 2:**
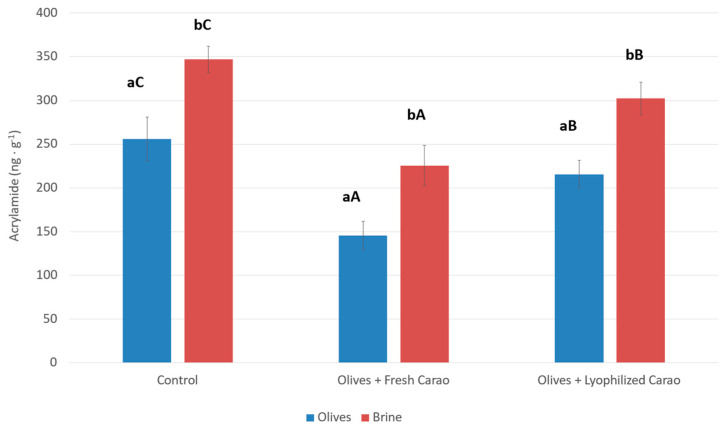
Acrylamide concentration (ng g^−1^) of California-style black olives of “Hojiblanca” variety after “Carao” addition. Different lowercase letters mean a statistically significant difference between the olives and the brine solution (Tukey’s test *p* < 0.05, n = 3). Different capital letters mean significant differences between the different “Carao” additions (Tukey test *p* < 0.05, n = 3).

For this, Californian-style table olives of “Hojiblanca” variety were used (Figure 2). As can be seen, the acrylamide content is significantly higher in the governing liquid than in the table olives. This may be because this molecule is hydrophilic and has an affinity for water. In this sense, the amount of acrylamide in table olives without “Carao” addition was 255.8 ng g^−1^, while in the governing liquid it was 347.3 ng g^−1^. The concentration of this toxic substance is in agreement with other studies on Californian-style table olives [61,62]. After the addition of the “Carao” to the governing liquid of Californian-style table olives, the concentration of acrylamide was significantly reduced compared to olives without addition. The fresh “Carao” addition showed the greatest activity in reducing acrylamide concentration, decreasing by 32.6% and 25.9% the amount of this compound in olives and brine solution, respectively. Instead, olives with lyophilized “Carao” were the least efficient for reducing acrylamide in this elaboration process. The reduction in this compound was of 11.8% and 10.7% in olives and brine solution, respectively. The reduction in this toxic substance after the addition of “Carao” may be due to the high content of phenolic compounds of this fruit. Phenolic extracts of olive leaf extract, white skin, and orange peel are efficient for reducing this compound, while Alpeorujo extract barely reduced acrylamide formation [33,63,64]. Other researchers [61,65] also indicated that the addition in cans of sterilized olives of certain herbs also reduced the acrylamide formation. Therefore, the addition of this fruit in Californian-style table olives, in addition to the high mineral content that it contributes to the final product, has the benefit of inhibiting the synthesis of acrylamide that is normally synthesized with the thermal sterilization treatments. Bassama et al. [64] also indicated that many natural antioxidant extracts were able to reduce acrylamide formation during the Maillard reaction. However, although there are many strategies to mitigate acrylamide content in table olives [4,6,28,61], until now there are no effective mitigation strategies to achieve acrylamide-free black table olives [65].

## 4. Conclusions

“Carao”, both fresh and freeze-dried, is a significant source of bioactive compounds, particularly those with antioxidant properties. It is also a valuable source of minerals, especially iron, making it suitable for the development of functional foods and food fortification. In this study, “Carao” was added to Californian-style black olives, a widely consumed product in the Mediterranean diet. However, some limitations of this work must be acknowledged; the two products investigated originate from completely different ecosystems, and “Carao” is not currently marketed, making its export economically unfeasible. In addition, it played an important role in the reduction in acrylamide, a substance with negative health effects formed in the heat treatment of food. This decrease was probably produced by the interaction of bioactive molecules, especially antioxidants on acrylamide, presenting a mitigating effect. Given the potential health benefits of enriching Mediterranean products such as olives with “Carao”, one viable approach would be to explore the domestication of “Carao” in Mediterranean regions, facilitating the commercialization of a new product: Californian olives enriched with “Carao”.

## Figures and Tables

**Table 1:** Total phenolic compounds and antioxidant activities in different treatments after “Carao” addition. TPCs: total phenol compounds; GAE: gallic acid; DPPH and ABTS: antioxidant method. Different lowercase letters mean a statistically significant difference between the different experimental treatments (Tukey’s test *p* < 0.05, n = 3).

Treatments	TPC (mg∙100 g^−1^) GAE	DPPH (mg∙100 g^−1^)	ABTS (mg∙100 g^−1^)
Fresh “Carao”	284.6 ± 2.3 a	878.5 ± 3.5 a	1034.2 ± 7.5 a
Lyophilized “Carao”	248.3 ± 4.3 b	721.2 ± 2.8 b	938.2 ± 1.8 b
Olives + brine	111.2 ± 5.2 c	541.2 ± 2.3 e	614.2 ± 5.3 d
Brine solution	12.3 ± 1.1 e	45.2 ± 1.2 g	23.2 ± 1.8 g
Olives + FeGluc	92.3 ± 3.1 d	378.1 ± 0.3 f	421.5 ± 1.2 e
Brine + FeGluc	12.9 ± 2.1 e	43.2 ± 0.1 g	39.2 ± 0.2 f
Olives + Fresh “Carao”	198.2 ± 3.2 a	1034.2 ± 2.0 a	1204.2 ± 3.5 a
Brine + Fresh “Carao”	141.3 ± 2.1 b	723.1 ± 1.0 c	921.3 ± 7.3 b
Olives + Lyophilized “Carao”	145.2 ± 2.1 b	803.2 ± 3.1 b	912.3 ± 2.3 b
Brine + Lyophilized “Carao”	112.1 ± 1.2 c	611.2 ± 2.1 d	804.2 ± 1.2 c

**Table 2:** Mineral composition in different table olive treatments after “Carao” addition. Different lowercase letters mean a statistically significant difference between the different experimental treatments (Tukey’s test *p* < 0.05, n = 3).

	Micronutrients (mg∙kg^−1^)							
Treatments	Copper (Cu)	Iron (Fe)	Manganese (Mn)	Zinc (Zn)	Calcium (Ca)	Magnesium (Mg)	Potassium (K)	Sodium (Na)
Fresh “Carao”	<0.5	3294.7 ± 121.6 a	18.2 ± 5.4 a	13.6 ± 8.6 ns	2094.0 ± 216.6 a	1365.2 ± 128.2 ns	2590.6 ± 28.3 a	165.7 ± 8.3 ns
Lyophilized “Carao”	<0.5	2101.5 ± 217.5 b	13.6 ± 3.6 b	12.5 ± 3.5	1570.0 ± 188.9 b	1421.6 ± 206.6	2103.4 ± 22.3 b	160.7 ± 8.3
Olives + brine	<0.5	120.4 ± 8.3 e	<0.5	10.9 ± 2.4 c	566.8 ± 25.6 d	247.3 ± 14.5 e	1094.3 ± 154.2 d	14,663.2 ± 254.5 b
Brine solution	<0.5	57.0 ± 6.4 f	<0.5	12.4 ± 3.1 c	139.4 ± 27.4 g	<0.5	189.7 ± 26.7 f	142.6 ± 9.3 e
Olives + FeGluc	<0.5	1017.9 ± 15.8 c	<0.5	11.4 ± 2.4 c	612.4 ± 32.3 e	300.1 ± 17.5 d	1425.5 ± 122.1 b	14,194.2 ± 136.4 b
Brine + FeGluc	<0.5	2202.5 ± 25.9 a	<0.5	19.0 ± 3.3 a	162.6 ± 14.6 f	<0.5	325.2 ± 22.1 e	270.2 ± 19.5 d
Olives + Fresh “Carao”	<0.5	893.5 ± 16.7 d	<0.5	11.2 ± 2.5 c	1385.0 ± 131.3 a	558.5 ± 21.4 c	1688.8 ± 101.4 a	19,434.7 ± 158.2 a
Brine + Fresh “Carao”	<0.5	1397.8 ± 235.1 b	5.0 ± 2.1 b	15.5 ± 2.2 b	1115.7 ± 264.1 b	748.8 ± 11.5 b	1387.8 ± 22.5 b	89.9 ± 15.6 f
Olives + Lyophilized “Carao”	<0.5	705.4 ± 114.7 d	<0.5	11.8 ± 1.2 c	1058.0 ± 111.2 b	517.3 ± 32.4 c	1464.8 ± 105.5 b	12931.5 ± 187.4 c
Brine + Lyophilized “Carao”	<0.5	1112.5 ± 146.3 c	7.4 ± 5.4 a	13.6 ± 2.5 c	865.3 ± 84.5 c	1003.8 ± 164.4 a	1149.6 ± 136.9 c	111.3 ± 11.3 f

ns = not significant.

## Data Availability

The original contributions presented in the study are included in the article, further inquiries can be directed to the corresponding author.
